# Australian Diabetes Foot Network: practical guideline on the provision of footwear for people with diabetes

**DOI:** 10.1186/1757-1146-6-6

**Published:** 2013-02-26

**Authors:** Shan M Bergin, Vanessa L Nube, Jan B Alford, Bernard P Allard, Joel M Gurr, Emma L Holland, Mark W Horsley, Maarten C Kamp, Peter A Lazzarini, Ashim K Sinha, Jason T Warnock, Paul R Wraight

**Affiliations:** 1 Australian Diabetes Foot Network, Australian Diabetes Society, Sydney, Australia; 2 Diabetic Foot Unit, Dandenong Hospital, Southern Health, Victoria, Australia; 3 Podiatry Department, Sydney Local Health District, Sydney, Australia; 4 Diabetes Centre, St Vincent’s Hospital, Darlinghurst, Sydney, Australia; 5 Vascular Surgery Unit, Western Hospital, Melbourne, Australia; 6 Podiatry Department, Royal Perth Hospital, South Metropolitan Health Service, Perth, Australia; 7 Diabetes & Endocrinology Service, Sunshine Coast Hospital & Health Service, Nambour, Australia; 8 Orthopaedics Department, Royal Prince Alfred Hospital, Sydney, Australia; 9 School of Clinical Sciences, Queensland University of Technology, Brisbane, Australia; 10 Cairns Diabetes Centre & James Cook University, Cairns, Australia; 11 Indigenous Diabetic Foot Program, Townsville, Australia; 12 Diabetic Foot Unit, Royal Melbourne Hospital, Victoria, Australia

**Keywords:** Footwear, Diabetes, Foot, Guideline, Australia

## Abstract

Trauma, in the form of pressure and/or friction from footwear, is a common cause of foot ulceration in people with diabetes. These practical recommendations regarding the provision of footwear for people with diabetes were agreed upon following review of existing position statements and clinical guidelines. The aim of this process was not to re-invent existing guidelines but to provide practical guidance for health professionals on how they can best deliver these recommendations within the Australian health system. Where information was lacking or inconsistent, a consensus was reached following discussion by all authors. Appropriately prescribed footwear, used alone or in conjunction with custom-made foot orthoses, can reduce pedal pressures and reduce the risk of foot ulceration. It is important for all health professionals involved in the care of people with diabetes to both assess and make recommendations on the footwear needs of their clients or to refer to health professionals with such skills and knowledge. Individuals with more complex footwear needs (for example those who require custom-made medical grade footwear and orthoses) should be referred to health professionals with experience in the prescription of these modalities and who are able to provide appropriate and timely follow-up. Where financial disadvantage is a barrier to individuals acquiring appropriate footwear, health care professionals should be aware of state and territory based equipment funding schemes that can provide financial assistance. Aboriginal and Torres Strait Islanders and people living in rural and remote areas are likely to have limited access to a broad range of footwear. Provision of appropriate footwear to people with diabetes in these communities needs be addressed as part of a comprehensive national strategy to reduce the burden of diabetes and its complications on the health system.

## Background

Diabetes related foot ulceration is a serious complication of diabetes and a significant risk factor for lower limb amputation [1-3]. There is limited data on the prevalence of foot ulcers in Australian populations with diabetes; however, the population based AusDiab study group (2003) described a community based foot ulcer prevalence of 2.1% [4]. A 2004 report from the Australian National Diabetes Information Audit (ANDIAB) indicated a foot ulcer prevalence of 1.9% amongst patients with diabetes attending specialist clinics [5]. A further 5.3% of the ANDIAB patient group reported a past history of foot ulceration and 77.2% of those with a current ulcer also had a past history of ulceration. Hospital admissions for diabetes related foot complications are frequent, with approximately 9,900 annual admissions reported by the Australian Institute of Health and Welfare (AIHW) for 2004/05 [6]. Given that 8.0% of all diabetes related deaths are directly attributable to foot ulcers and that the presence of a foot ulcer increases the risk of initial amputation fivefold, all health professionals involved in the care of people with diabetes have a responsibility to identify and mitigate causes of foot ulceration [6,7].

Whilst the presence of peripheral neuropathy is the leading risk factor for diabetes related foot ulceration, a pivotal event, such as trauma from footwear, is also needed for most ulcers to occur [1]. Rubbing from footwear was identified as the definite cause of 35.0% of foot ulcers reviewed as part of a prospective study conducted in the United Kingdom [8]. Furthermore, the follow-up of 472 patients at The Royal Prince Alfred Hospital Diabetes Centre (NSW, Australia) identified that 54.0% of all foot ulcers that developed in this group, could be directly attributed to trauma from footwear [9].

The purchase and wearing of appropriate footwear is therefore an important process for all individuals with diabetes, especially for those who demonstrate loss of protective sensation from peripheral neuropathy. This patient group, who are unable to feel pressure and/or pain caused by inappropriate or ill-fitting shoes are more likely to develop blisters, callus and corns. These early complications are pathological and are warning signs that require prompt intervention if ulceration and potential amputation are to be avoided. The simple measure of wearing appropriately fitted or prescribed footwear has been shown to significantly reduce plantar foot pressures, therefore decreasing the likelihood of developing callus and ultimately ulceration [10,11].

Health professionals involved in the care of people with diabetes need to define the individuals’ level of risk for developing foot complications and thus tailor footwear advice accordingly. Risk stratification is determined following a basic foot assessment, which includes evaluation for the presence of peripheral neuropathy, peripheral arterial disease and foot deformity [12-14]. In addition to the foot assessment, other factors that should to be considered include the individuals’ activity levels, occupation and level of mobility. For example, individuals who are employed on a work site may require steel capped footwear in order to comply with industry standards. Risk stratification should be re-assessed and upgraded on a yearly basis, given the potential for progression and development of new risk factors over time.

Guidelines from the National Health and Medical Research Council (NHMRC) cite appropriate footwear as one of the key strategies in preventing foot ulceration [13,14]. This document therefore builds on the NHMRC’s recommendation by providing practical guidelines to Australian health professionals; informing them on how to provide appropriate footwear for people with diabetes, based on their risk stratification for developing a foot ulcer (Tables 1 and 2).

**Table 1:** Footwear recommendations for people with diabetes based on their risk stratification for developing foot ulceration

**Category**	** Foot assessment**	**Recommendations**
** Low**	No peripheral neuropathy	Off the shelf footwear is likely to be appropriate.
	No peripheral arterial disease	Encourage clients to have their feet measured and professionally fitted.
	Normal foot shape	Encourage clients to wear footwear that meets the criteria in Table 2.
	No history of amputation	
** At Risk**	Peripheral neuropathy	Off the shelf footwear is likely to be appropriate.
	AND / OR	Encourage clients to have their feet measured and professionally fitted.
	Peripheral arterial disease	Encourage clients to wear footwear that meets the criteria in Table 2.
	Normal foot shape	Footwear must be worn at all times to protect feet from injury.
	No history of amputation	Fit footwear in the afternoon to ensure any dependent oedema is accommodated.
		New footwear should be worn in gradually.
		Check feet regularly for signs of trauma when wearing in new shoes.
** High**	Abnormal foot shape, including History of amputation	Footwear assessment by an appropriately trained health professional recommended.
		Medical grade footwear and custom moulded foot orthoses will generally be required.
		Foot orthoses to be supplied prior or together with prescribed footwear.
		Footwear must be worn at all times to protect feet from injury.
		Fit footwear in the afternoon to ensure any dependent oedema is accommodated.
		New footwear should be worn in gradually.
		Check feet regularly for signs of trauma when wearing in new shoes.

**Table 2:** Shoe features that clients should be aware of when purchasing footwear

** Shoe features**	**Criteria for choosing protective footwear features**
**Uppers**	These should be made from leather or a combination of materials (such as those used in sports shoes) with smooth inner lining and no bulky seams at the toe area.
**Correct length**	1 cm from end of longest toe when client is standing.
**Correct depth**	Accommodate the toes without causing pressure.
**Correct width**	The sides of the shoe should not bulge over the last (sole) when worn.
**Low heels**	Less or equal to 2 cm.
**Fastening**	Adequate fastening such as laces or Velcro to keep the foot from sliding forward.
**Cushioned outer and inner soles**	Approximately 0.5-1 cm thick under the forefoot.
**Enclosed heel**	Open backed shoes can result in injury to the skin around the heel and usually require the individual to claw their toes in order to keep them on, also increasing risk of ulceration.
**Soles**	Non slip.

### Low risk: footwear for people with no risk factors for ulceration

Individuals with no identifiable risk factors (no peripheral neuropathy, peripheral arterial disease, foot deformity or history of amputation) on foot assessment are at low risk for developing foot ulceration (Table 1). A commonsense approach to footwear selection and use is advisable. Individuals can usually be safely accommodated in a wide range of off the shelf footwear, provided they are correctly fitted and appropriate for the activity to be undertaken. Table 2 summarises the features that individuals should be aware of when purchasing footwear.

### At risk: footwear for people with peripheral neuropathy and/or peripheral arterial disease

Individuals with peripheral neuropathy and/or peripheral arterial disease on foot assessment are ‘at risk’ of developing foot ulceration (Table 1) [15]. Correctly fitted footwear, especially with regards to length and width, is essential. This is especially important for individuals with peripheral neuropathy who may have a tendency to purchase poorly fitting shoes in order to stimulate some sensory feedback [16]. The majority of individuals in this category should be able to be accommodated in off the shelf footwear. The criteria for selecting appropriate footwear (Table 2) should again be applied and individuals should be advised to have themselves fitted in the afternoon in order to accommodate any dependent oedema. New shoes should be worn in, with wearing time gradually increased over a 1 to 2 week period. When shoes are removed, the individual should check their feet for signs of pressure, trauma and ulceration. Footwear should also be checked daily, inspecting for signs of wear and tear, and ensuring that there is no foreign object within the shoe or penetrating through the sole.

### High risk: footwear for people with abnormal foot shape or history of amputation

Individuals assessed as having an abnormal foot shape, including previous amputation, are at high risk of developing foot ulceration (Table 1) [16]. The severity of the foot deformity will directly influence footwear prescription but in general the requirement will be for medical grade footwear and custom moulded foot orthoses. Custom-made medical grade footwear and orthoses should be considered for individuals with severe deformities and additional modifications, such as rocker soles (for pressure relief) may be indicated. Once again, the prescribed footwear should meet the criteria as set in Table 2. Medical grade footwear and custom moulded foot orthoses should be reviewed for wear and be replaced as required. A referral to a health professional with the knowledge and skills for prescribing appropriate footwear is recommended.

Individuals with a previous foot ulcer are at high risk of developing further ulcers in the future [17]. Even though a past ulcer does not define the footwear needs of the individual, it should be seen as an alert that leads the individual to being prioritised in acquiring prompt access to appropriate footwear.

### Orthoses

Foot orthoses may be ‘non-moulded’ and designed to provide cushioning or ‘custom-moulded’ to additionally accommodate the shape of the foot and reduce plantar pressures [18]. Custom moulded foot orthoses are indicated for individuals with significant deformity and plantar prominences (such as under the metatarsal heads), especially if there is a history of plantar foot ulceration [18]. Both should be supplied by an appropriately trained health professional and be fabricated for inclusion in the footwear at, or prior to prescribing the footwear. Orthoses placed into existing footwear or shoes not designed to accommodate an orthosis, may render the footwear too tight, placing the foot at risk of pressure and ulceration. Foot orthoses should be regularly reviewed to ensure they are providing adequate pressure relief and are not excessively worn.

### Access to medical grade footwear

Individuals who require medical grade footwear may find the cost prohibitive. Those who cannot afford medical grade footwear may be eligible for government funding through one of the state/territory based schemes. Health care professionals need to be aware of what footwear schemes exist in their area and refer appropriate individuals for consideration. Websites for state based schemes for the provision of footwear and orthoses are provided in Table 3. Each funding body will have its own application process and eligibility criteria. Some states also have funding available for individuals who have undergone amputation, however, criteria such as level of amputation is often applied. The Department of Veterans’ Affairs funds medical grade footwear for ‘Gold’ card holders. However, financial authorisation is required prior to referral and the footwear can only be provided by contracted suppliers, who are accessible through footwear prescribers.

**Table 3:** State based schemes that may provide financial assistance to clients requiring footwear and/or orthoses

**Footwear scheme (State)**	**Footwear scheme website**
ACT Equipment Scheme (Australian Capital Territory)	http://health.act.gov.au/c/health?a=sp&pid=1059610195
Enable (New South Wales)	http://www.enable.health.nsw.gov.au/
Territory Independence and Mobility Equipment Scheme (Northern Territory)	http://www.health.nt.gov.au/Aged_and_Disability/Subsidies/TIME_Scheme/index.aspx
Medical Aids Subsidy Scheme (Queensland)	http://www.health.qld.gov.au/mass/
Lousia DaCosta Trust (South Australia)	Lousia DaCosta Trust. Email: info@dacosta.net.au
	The Wyatt Benevolent Institution. http://www.wyatt.org.au
Community Equipment Scheme (Tasmania)	http://www.dhhs.tas.gov.au/service_information/disability/community_equipment_scheme
Aids and Equipment	http://www.vic.gov.au/health-community/disability-services/aids-equipment.html
Program (Victoria)	
Community Aids and Equipment Program (Western Australia)	http://www.disability.wa.gov.au/DSCWR/_assets/main/Promotional/Documents/PDF/CAEP_BROCHURE.PDF

### Aboriginal and Torres Strait Islanders and geographically remote individuals

Individuals in rural and remote areas may have a limited range of footwear options available to them. Many general shoe stores servicing these communities do not have foot measuring devices or skilled staff to assist with fitting. Inequities also exist in the availability of medical grade and custom-made medical grade footwear providers in rural and remote areas. Often smaller communities will only have an occasional visiting service.

Aboriginal and Torres Strait Islanders who live in remote locations may have no access to footwear and potentially may not understand the health benefits of wearing particular footwear even when they have diabetes. Recent pilot programs in remote locations in Queensland have demonstrated that Aboriginal and Torres Strait Islanders can be encouraged to wear footwear to protect their feet [J. Warnock, Personal Communication 2010]. These multi-disciplinary programs provide footcare education, diabetic foot screening, podiatric interventions and appropriately fitted footwear. Different environmental and cultural circumstances may require a compromise to be reached, whereby footwear such as reef sandals, that are not generally recommended for individuals with diabetes, may be considered better than no footwear at all.

## Conclusions

Individuals with diabetes need consistent and ongoing education from health care providers regarding the role of footwear and the type most appropriate to their level of risk for ulceration. Access to government funding for footwear is variable across the States and Territories and long waiting times can be experienced. Individuals who cannot afford medical grade footwear, custom-made medical grade footwear and/or foot orthoses, despite being assessed as medically requiring these devices, should be provided with assistance from health professionals to apply for funding via government subsidised schemes.

### List of definitions

**Abnormal foot shape:** An abnormal foot shape is one that cannot be accommodated in regular, off the shelf footwear. This includes, but is not limited to feet with: hallux valgus, clawed/hammer toes, severe pes-planus or cavus foot type, abnormally wide feet, partial foot amputation or chronic Charcot deformity.

**Custom-made medical grade footwear:** Custom-made medical grade footwear (or bespoke footwear) has the features of medical grade footwear but is manufactured specifically for one individual, as the individual cannot be safely accommodated in non custom-made footwear. Multiple measurements, impressions or a mould is generally required for manufacture.

**Custom moulded foot orthoses / insoles:** Custom moulded foot orthoses or insoles are manufactured using an impression of the foot and are generally comprised of multiple layers of different materials. They are designed to conform to the shape of the foot, providing cushioning and redistribution of plantar pressure.

**Medical grade footwear:** Medical grade (or therapeutic) footwear is footwear that meets the specific needs of the client, on the basis that they provide extra depth, multiple width fittings and features designed to accommodate a broader range of foot types. Other features may include modified soles, fastenings and smooth internal linings. This type of footwear is usually available at specialty shoe shops.

**Off the shelf footwear:** Readily available comfort or sports shoes that have not been modified.

**Peripheral Arterial Disease (PAD):** Absence of both pedal pulses, past intervention for PAD or established history of PAD.

**Peripheral neuropathy:** Sensory loss that prevents the individual feeling a 10 g monofilament.

## Competing interests

The authors have no relevant conflict of interest to disclose.

## Authors’ contributions

This manuscript was conceived, developed and approved by the Australian Diabetes Foot Network (ADFN). The ADFN is a working group of the Australian Diabetes Society and members include health professionals from endocrinology, podiatry, nursing/diabetes education, indigenous health, orthopaedic surgery and vascular surgery. Each member of the ADFN is an author of this manuscript, contributed to its development, agrees with its recommendations and approved the final manuscript.

## References

[B1] Reiber GE Vileikyte L Boyko EJ del Aguila M Smith DG Lavery LA Boulton AJ Causal pathways for incident lower–extremity ulcers in patients with diabetes from two settings Diabetes Care 1999 22 157 62 10.2337/diacare.22.1.157 10333919

[B2] Apelqvist J Bakker K van Houtum WH Schaper NC Practical guidelines on the management and prevention of the diabetic foot: based upon the International Consensus on the Diabetic Foot (2007): prepared by the International Working Group on the Diabetic Foot Diabetes Metab Res Rev 2008 24 1 S181 S187 10.1002/dmrr.848 18442189

[B3] Frykberg RG Epidemiology of the diabetic foot: ulcerations and amputations Adv Wound Care 1999 12 139 41 10655794

[B4] Tapp RJ Shaw JE de Courten MP Dunstan DW Welborn TA Zimmet PZ Foot complications in type 2 diabetes: an Australian population-based study Diabet Med 2003 20 105 113 10.1046/j.1464-5491.2003.00881.x 12581261

[B5] National Association of Diabetes Centres (NADC) Australian national diabetes information audit and benchmarking (ANDIAB) 2004. NADC, ACT 2005 http://www.health.gov.au/internet/main/publishing.nsf/Content/pq-diabetes-pubs

[B6] Australian Institute of Health & Welfare (AIHW) Diabetes: Australian Facts 2008 2008 Canberra: Australian Govt http://www.aihw.gov.au/publications/cvd/daf08/daf08.pdf

[B7] Davis WA Norman PE Bruce DG Davis TM Predictors, consequences and costs of diabetes-related lower extremity amputation complicating type 2 diabetes: the Fremantle Diabetes Study Diabetalogia 2006 49 2634 41 10.1007/s00125-006-0431-0 17001469

[B8] MacFarlane RM Jeffcoate WJ Factors contributing to the presentation of diabetic foot ulcers Diabet Med 1997 14 867 870 10.1002/(SICI)1096-9136(199710)14:10<867::AID-DIA475>3.0.CO;2-L 9371480

[B9] McGill M Molyneaux L Yue DK Which diabetic patients should receive podiatry care? An objective analysis Intern Med J 2005 35 451 6 10.1111/j.1445-5994.2005.00880.x 16176466

[B10] Pitei D Watkins PJ Foster AVM Edmonds ME Do new EVA moulded insoles or trainers efficiently reduce the high foot pressures in the diabetic foot? Diabetes 1996 1A 389

[B11] Cavanagh PR Ulbrecht JS Caputo GM New developments in the biomechanics of the diabetic foot Diabetes Metab Res Rev 2000 16 Suppl 1 S6 S10 11054880 10.1002/1520-7560(200009/10)16:1+<::aid-dmrr130>3.0.co;2-z

[B12] International Working Group on the Diabetic Foot (IWGDF) / Consultative Section of International Diabetes Federation (IDF) International Consensus on the Diabetic Foot & Practical Guidelines on the Management and the Prevention of the Diabetic Foot 2007 Amsterdam, the Netherlands: IWGDF

[B13] Australian Centre for Diabetes Strategies Identification and management of diabetic foot disease, in National evidenced based guidelines for the management of Type 2 Diabetes Mellitus 2005 Sydney: Diabetes Australia Guideline Development Consortium

[B14] National Health & Medical Research Council (NHMRC) Guideline National evidence-based guideline on prevention, identification and management of foot complications in diabetes (Part of the guidelines on management of type 2 diabetes) 2011 Melbourne: Baker IDI Heart & Diabetes Institute http://www.nhmrc.gov.au/guidelines/publications/subject/Diabetes

[B15] Boyko EJ Ahroni JH Stensel V Forsberg RC Davignon DR Smith DG A prospective study of risk factors for diabetic foot ulcer The Seattle Diabetic Foot Study. Diabetes Care 1999 22 7 1036 42 10.2337/diacare.22.7.1036 10388963

[B16] Cavanagh PR Therapeutic footwear for people with diabetes Diabetes Metab Res Rev 2004 20 Suppl 1 S1 S55 15150815 10.1002/dmrr.435

[B17] Murray HJ Young MJ Hollis S Boulton AJ The association between callus formation, high pressures and neuropathy in diabetic foot ulceration Diabet Med 1996 13 11 979 982 10.1002/(SICI)1096-9136(199611)13:11<979::AID-DIA267>3.0.CO;2-A 8946157

[B18] Bus SA Ulbrecht JS Cavanagh PR Pressure relief and load redistribution by custom-made insoles in diabetic patients with neuropathy and foot deformity Clin Biomech 2004 19 6 629 38 10.1016/j.clinbiomech.2004.02.010 15234488

